# Epigenetic modifications of histones in cancer

**DOI:** 10.1186/s13059-019-1870-5

**Published:** 2019-11-20

**Authors:** Zibo Zhao, Ali Shilatifard

**Affiliations:** 1 Department of Biochemistry and Molecular Genetics, Northwestern University Feinberg School of Medicine, Simpson Querrey 7th Floor 303 E. Superior Street, Chicago, IL 60611 USA; 2 Simpson Querrey Center for Epigenetics, Northwestern University Feinberg School of Medicine, Chicago, IL 60611 USA

## Abstract

The epigenetic modifications of histones are versatile marks that are intimately connected to development and disease pathogenesis including human cancers. In this review, we will discuss the many different types of histone modifications and the biological processes with which they are involved. Specifically, we review the enzymatic machineries and modifications that are involved in cancer development and progression, and how to apply currently available small molecule inhibitors for histone modifiers as tool compounds to study the functional significance of histone modifications and their clinical implications.

## Introduction

In eukaryotic cells, DNA is packed as chromatin whose functional units are nucleosomes. Each nucleosome is composed of an octamer of four core histones (H3, H4, H2A, and H2B), around which is wrapped 147 base pairs of DNA [1]. The globular regions of the histones form the core of the nucleosome, while the N-terminal tails protrude from the nucleosomes and are enriched with a variety of posttranslational modifications (PTMs). PTMs can also occur on the lateral surface of the nucleosome core regions of histones that are in contact with the DNA [2], with both tail and core modifications influencing the chromatin structure by altering the net charge of histones, by altering inter-nucleosomal interactions, and by facilitating the recruitment of specific proteins such as bromo-, chromo-, Tudor, PWWP, MBT, and PHD domain-containing proteins [1].

Histone modifications, and the enzymes implementing them, can contribute to chromatin compaction, nucleosome dynamics, and transcription. These modifications can be implemented in response to intrinsic and external stimuli. Dysregulation of these processes can shift the balance of gene expression and are therefore frequently observed in human cancers, either by gain or loss of function, overexpression, suppression by promoter hypermethylation, chromosomal translocation, or mutations of the histone-modifying enzymes/complexes or even the modification site of the histone [2–4]. Indeed, mutations in chromatin-bound proteins are among the top frequently mutated targets in cancer [5]. The dysregulation of certain chromatin-associated proteins may act as drivers in certain types of cancer [6, 7]. Consequently, abnormal cellular proliferation, invasion, and metastasis and chemoresistance may occur during disease progression [8]. However, there is still a substantial base of knowledge that needs to be gained in order to define the roles of histone modifications and its enzymatic machinery during development and disease settings.

This review focuses on the recent progress in our understanding of histone modifications in mammals, highlighting the mechanisms of PTMs in cancer with the availability of new assays, techniques and inhibitors for fine mapping the modifications genome-wide and the potential to use in the treatment of cancers. We will define what marks are epigenetic, and why and how the balance is maintained between different modifications for proper regulation of gene expression. We will also address the histone modifications in cancer as biomarkers of cancer progression and/or prognosis.

### Histone modifications, modifiers, and their functions in development and cancers

**Fig. 1:**
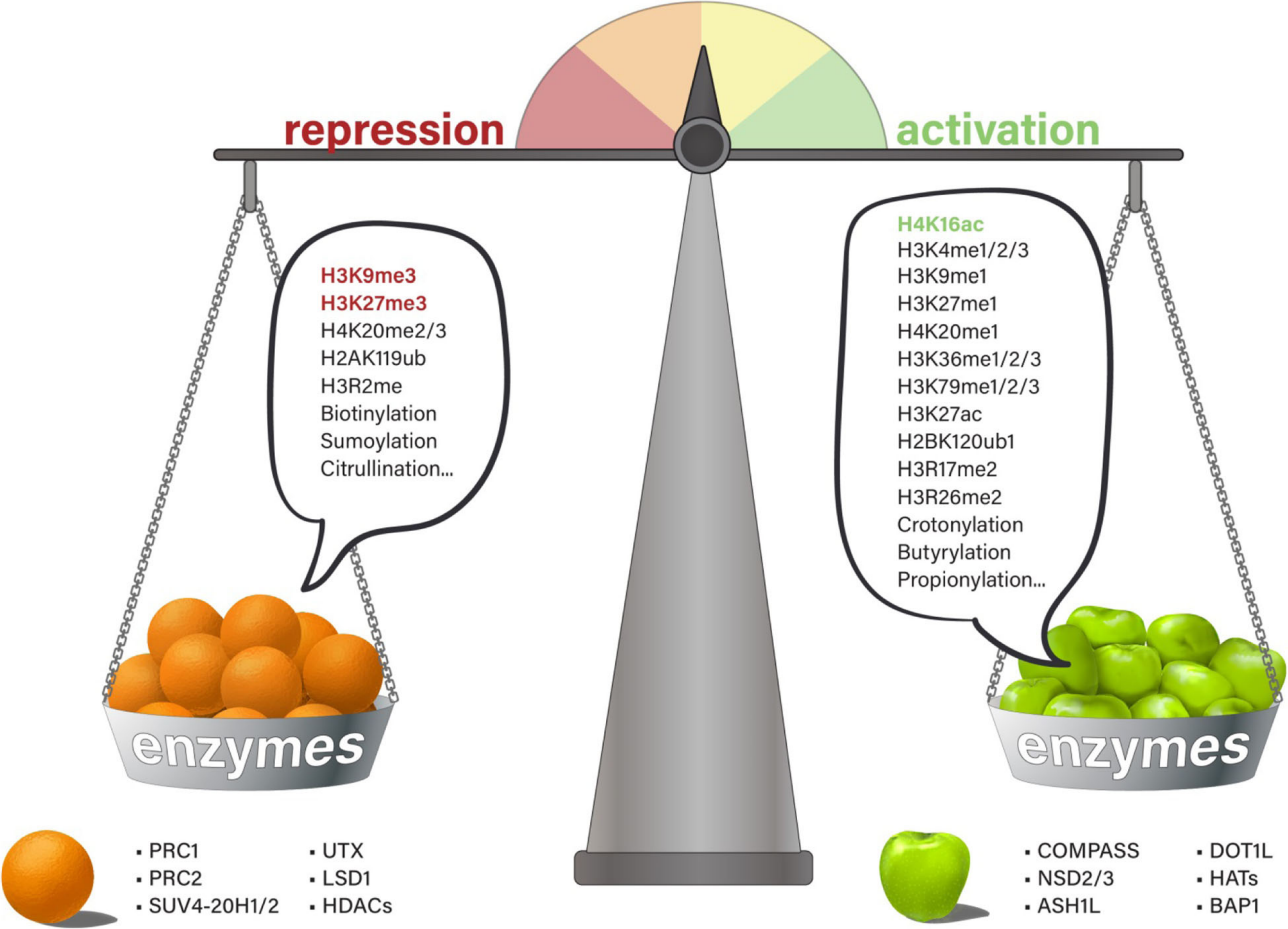
Balanced states of transcription maintained by the versatile chromatin proteins and histone modifications. The balanced states of transcription are maintained by the chromatin modifiers and histone modifications. The histone-modifying enzymes are depicted as apples (activation) and oranges (repression) in the two weighing pans respectively. The chromatin states are maintained and balanced by a number of activation marks and repression marks. Histone marks highlighted in bold are considered to be hallmarks of euchromatin (H4K16ac) and heterochromatin (H3K9me3 and H3K27me3) respectively

**Fig. 2:**
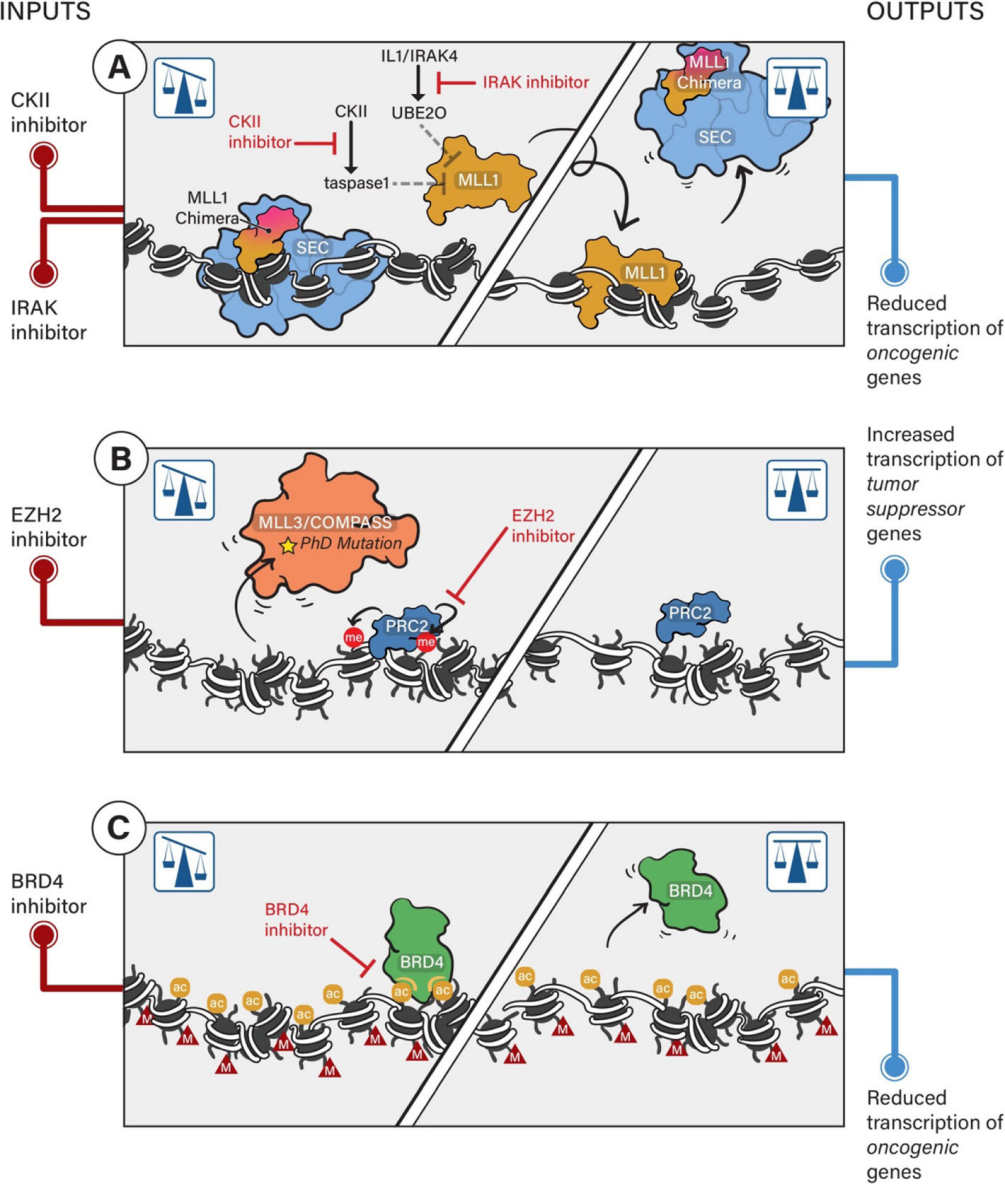
Pharmacological restoration of the epigenetic balance of gene expression in human cancers. **a** MLL translocation and SEC promote the leukemogenesis in MLL-rearranged leukemia. Enhancing the wild-type MLL1 recruitment to chromatin by hijacking the IL1/IRAK4 and CKII/tasapse1 pathways displaces the MLL chimera and SEC and inhibits leukemogenesis. **b** MLL3 mutation in the PHD leads to the loss of function of MLL3/COMPASS and decreased enhancer H3K4 methylation. EZH2 inhibition by small molecules (e.g., GSK-126) inhibits EZH2 enzymatic activity and decreases H3K27 methylation to restore the tumor suppressor gene expression. **c** H3K27M mutation leads to the global increase of H3K27 acetylation and aberrant gene expression. Inhibition of BRD4 by small molecules (e.g., JQ-1) displaces the protein from chromatin and restores the normal-like gene expression and inhibits DIPG from progression

Transcription activation and repression are controlled by an array of histone modifiers and chromatin-bound proteins. A balance between specific modifications and modifiers are maintained at the steady state of the cell to maintain the chromatin structure, execute the proper gene expression program, and control the biological outcome (Fig. 1). Once the balance is disrupted, cell phenotypes may be altered and primed for disease onset and progression [9–11]. Therefore, understanding the functions of the key regulators of histone modifications will help us to develop chemical probes to maintain the homeostasis and restore the balanced state of the cell (Fig. 2).

We primarily focus this review on methylation, acetylation, and ubiquitination of the PTMs associated with the development and cancers. The other types of modifications including those that are newly identified will also be briefly discussed towards the end of the review. The major types of modifications of histones on tails or within the nucleosome core that are discussed in this review are summarized in Table 1.

**Table 1:** Chromatin modifiers, binding factors and functions of selected PTMs on histones

Histones	Modification	Modifiers		Binding factors	Functions	Reference
H2A	K119Ub1	RING1A/B	BAP1, USP16, USP21, 2A-DUB, USP3, USP22		Transcriptional repression	[12, 13]
H2AX	S139p (γH2AX)	ATM, ATR, DNA-PK	PP2A, Wip1, PP6 and PP4	MDC1, CRB2	DNA repair	[14]
H2B	K120Ub1	RNF20, RNF40	USP3, USP7, USP22		Transcriptional activation, DNA damage response	[15]
H3	K4me1/2/3	SET1A/B, MLL1-4	KDM1A/B, KDM2A/B, KDM5A/B/C/D	BRWD2/PHIP, MLL,TAF3, CHD1,RAG2, BPTF, PHF2/6/8, JMJD2	Transcriptional activation	[16–18]
	K9me1/2/3	Suv39H1/2, G9a, GLP, SETDB1	KDM1A, KDM3A/B, JMJD1C, KMD4A/B/C/D	HP1, ATRX	Transcriptional activation (K9me1), repression (K9me2/3), X-inactivation and imprinting (K9me2)	[19]
	S10p	Aurora B, MSK/RSK/Jil-1	PP2A, PP1	14–3-3ζ	Mitosis, meiosis, transcriptional activation	[20]
	R26me2	CARM1	PADI4		Transcriptional activation	[21]
	K27 ac	CBP/p300	HDACs	BRDs	Transcriptional activation	[22]
	K27me1/2/3	EZH1/2	KDM6A/B, KDM7A, PHF8	EED, PC, CBX7	Transcriptional activation (K27me1); Transcriptional silencing, X-inactivation, bivalent genes/gene poising (K27me2/3)	[23]
	K36me1/2/3	NSD1–3, SETD2/3, ASH1L, SETMAR, SMYD2	KDM2A/B, KDM4A/B/C/D, JHDM1A	ZYMND11, PHF19, LEDGF	Transcriptional elongation, repression, DNA repair	[19]
	K79me1/2/3	DOT1L	?	p53BP1	Transcriptional activation	[24]
H4	K20me1	PR-Set7	LSD1n	CRB2, p53BP1	Transcriptional activation	[25, 26]
	K20me2/3	SUV4-20H1/2	LSD1n, DPY-21	CRB2, p53BP1, JMJD2	Transcriptional silencing, Heterochromatin	[25, 27]
	K16 ac	MOF	HDACs, Sirt2	BRDs	Transcriptional activation, DNA repair	[28, 29]

#### Methylation

Histone methylation is a dynamic process with key roles in development and differentiation [30, 31]. For instance, H3K4 methyltransferases play crucial roles on Hox gene regulation during developmental stage [32, 33]. Aberrant levels of histone methylation are likely to play a causal role in tumorigenesis. The outcomes of methylation on histones are highly context dependent and can be associated with different gene expression status. Histone methylation is intimately associated with transcriptional regulation by influencing chromatin architecture, recruiting transcriptional factors, interacting with initiation and elongation factors, and affecting RNA processing [34].

Histone methylation takes place on the side-chain nitrogen atoms of both lysine and arginine residues, most heavily on histone H3 followed by H4 [35]. Multiple methylation states exist for both lysine and arginine methylation, and these can elicit different outcomes for transcriptional regulation. Lysine can be mono-, di-, or trimethylated by six major classes of histone lysine methyltransferase complexes (KMT1-6) [36]. The KMT1 family contains at least four members in mammals including SUV39H1/2, G9a, GLP, and SETDB1, with H3K9 as the substrate for methylation [37, 38]. The KMT2 family enzymes are found within the macromolecular complex called complex of proteins associated with Set1 (COMPASS) and deposit mono-, di-, or trimethyl marks on H3K4 [16–18]. The KMT3 family contains NSD1, NSD2 (WHSC1), and NSD3 (WHSC1L1) and primarily methylates H3K36 [39]. The KMT4 family has DOT1L as the sole member, which implements H3K79 methylation [24, 40]. The KMT5 family comprises the PR-Set7 and SUV4-20H1/2, which implement H4K20 monomethylation and di-/trimethylation, respectively [41]. The KMT6 family includes the functionally redundant enzymes EZH1 and EZH2 for H3K27 mono-, di-, and trimethylation [23].

Lysine methylation has been known to be a reversible process since the discovery of the lysine demethylase LSD1 [42]. There are at least six families of histone lysine demethylases with both unique and overlapping functions. The KDM1 family includes LSD1 (KDM1A) and LSD2 (KDM1B), both of which can demethylate H3K4me2/me1 but not H3K4me3 [42, 43]. Moreover, LSD1 can also work on H3K9 demethylation through the switching from its repressive complex with CoREST interaction to an activating complex with androgen receptor (AR) interaction [19, 44–46]. All other family members of lysine demethylases harbor the Jumonji (JmjC) domain, which due to the different chemistry involved have the potential for removing the trimethyl mark, unlike the LSD family. JHDM1A (KDM2A) and JHDM1B (KDM2B) belong to the KDM2 family with activities towards H3K36me2/me1 and H3K4me3 [19]. JHDM1A was the first JmjC domain-containing demethylase identified [47]. The KDM3 family comprises KDM3A, KDM3B, and JMJD1C, with demethylase activities for H3K9me2/me1 [19]. The KDM4 family includes KDM4A, KDM4B, KDM4C, and KDM4D, with diverse demethylase activities towards H3K9me3/me2 and H3K36me3/me2. The KDM5 family contains KDM5A, KDM5B, KDM5C, and KDM5D, all of which can demethylate H3K4me3/me2. KDM6 family includes UTX (KDM6A), JMJD3 (KDM6B), and UTY. UTX and JMJD3 are specific for H3K27me3/me2, while the Y-linked paralog, UTY, has little catalytic activity. Several of the KDMs have been considered as contributing factors for the development of multiple cancers, and thus postulated to be potential drug targets. KDM inhibitors could be valuable for both elucidating their cellular functions and as potential therapeutics [48–50].

The most well-characterized methylation marks on lysine residues associated with transcriptional activation include H3K4 [51], H3K36 [39], and H3K79 [24], and transcriptional repression-associated methylations occur on H3K9 [37], H4K20 [41], and H3K27 [52] (Fig. 1). Notably, the co-occurrence of large regions of H3K27 methylation harboring smaller regions of H3K4 methylation marks constitutes the “bivalent domains,” which are thought to be important for maintaining pluripotency by silencing developmental genes in embryonic stem cells (ESCs) while keeping them poised for activation during developmental stage [53–55]. Altering the balance of these histone modifications of gene expression may contribute to the pathogenesis of cancers [9, 10].

Histone H3K4 methylation is implemented by methyltransferases in the COMPASS family including SET1A, SET1B, and MLL1-4 at enhancers and promoters [16, 54, 56–60]. Different subunits of COMPASS have also been shown to regulate H3K4 di- and/or trimethylation including WDR5, Ash2L, RbBP5, and Dyp30 which are subunits shared by all COMPASS family members [61, 62]. SET1A and SET1B primarily trimethylate histone H3K4 at promoters [16, 63], albeit the majority of SET1B is localized in the cytoplasm [57]. Interestingly, the oncogenic function of SET1A has been implicated in breast cancer metastasis, lung cancer, and colorectal cancer tumorigenesis through both methylation of histones and the non-histone substrate YAP respectively [64, 65]. MLL1 and MLL2 implement di- and trimethylation at promoters and/or Polycomb response elements (PRE), and MLL2 can also methylate H3K4 at both promoters of bivalent genes and enhancers [17, 54, 59]. Interestingly, the reconstituted MLL1 SET domain with WRAD complex allows it to mono-, di-, and trimethylate H3K4 in vitro [61, 66], although the monomethylation activity of MLL1 in vivo was not demonstrated so far. MLL3 and MLL4 are capable for the monomethylation of H3K4 at enhancers [67]. The methylation kinetics by MLL1 core complex demonstrated in the in vitro reconstitution assays suggests that the di- or trimethylation by SET1A, SET1B, MLL1, and MLL2 may not require the monomethylation by MLL3 and MLL4. This was also supported by the distinct genomic localization of different COMPASS methyltransferases demonstrated in ChIP-seq of these factors [58, 59, 67, 68].

Although structures of COMPASS family of H3K4 methyltransferases have been resolved recently [61, 69, 70], small molecule inhibitors that directly inhibit the enzymatic activities are still unavailable. Development of these inhibitors would not only serve as molecular tools to dissect the detailed functions but also contribute to clinical treatment of various cancers with aberrant activities or expression of COMPASS methyltransferases. In addition to the well-studied methyltransferase activity of the COMPASS family, recent efforts have been devoted to investigating the catalytic independent roles of COMPASS methyltransferases (and the same approach could be applied to other types of histone modifiers) [58, 71–74]. For instance, the requirement of SET1A in ESC proliferation and self-renewal is unaffected by removal of the catalytic SET domain, while the SET domain is required for proper differentiation [58]. Likewise, SET1B, independent of its SET domain, is essential for suppressing ADIPOR1 signaling in the cytoplasm for eliciting tumorigenic effect [57]. Given the importance of SET1B-ADIPOR1 signaling in triple negative breast cancer (TNBC), AdipoRon, the ADIPOR1 agonist, has been proposed as a novel therapeutic strategy for clinical treatment of TNBC [57].

MLL1 is frequently mutated through translocation with other oncogenic partners in acute myeloid and lymphoid leukemia (AML and ALL), accounting for ~ 80% of childhood leukemia and 5–10% of adult leukemia [75]. The chimeric proteins lack the catalytic SET domain of MLL1 and drive leukemogenesis. Recently, we identified strategies for the treatment of MLL-rearranged leukemia via stabilizing the wild-type copy of MLL to attenuate the aberrant transcription mediated by MLL fusion proteins and their oncogenic co-factor, the super elongation complex (SEC) [76, 77] (Fig. 2a). These studies also indicate that not only the catalytic activities but also the protein levels/protein turnover determine the outcome of their activities. Nonetheless, completely knocking out the oncogenic fusion proteins still remains a hard-to-target issue in MLL-rearranged leukemia.

MLL3 and MLL4 are both found to be highly mutated in cancer [4, 10, 18, 78]. MLL3 has a mutation hot spot at the plant homeodomain (PHD) cluster, whereas MLL4 mutations are more evenly distributed throughout the protein [10, 18]. Our recent study documented that mutations within the MLL3 PHD cluster disrupt its interaction with the BAP1 tumor suppressor and correlates with poor patient survival [10]. Since MLL3 and MLL4 catalytic activity is dispensable for development and enhancer RNA synthesis [72, 73], it will be important to investigate catalytic and non-catalytic tumor suppressor roles of these proteins.

The histone H3K4me3 mark can help recruit the chromatin remodeling factors CHD1 [79] and BPTF [80], chromatin remodelers which can help open chromatin. In addition, our laboratory discovered that BRWD2/PHIP may recognize H3K4 methylation marks through a CryptoTudor domain adjacent to a bromodomain, suggesting synergy between acetylation and methylation in transcription regulation by this protein [81]. Pharmacologically targeting the catalytic activity of COMPASS methyltransferases, the protein-protein interactions (PPI) between key COMPASS subunits, or the binding of proteins to methylated H3K4, can each be harnessed to further facilitate the understanding of the downstream events and open new therapeutic approaches for cancer treatment. PPI disruptors of the Menin-MLL interaction, namely MI-463, MI-503, and M-525 [82, 83] and OICR-9429 for the WDR5-MLL interaction [84], have been developed with the hope of treating MLL-rearranged and CEBPA mutant leukemia. A complete list of compounds discussed in this review is listed in Table 2.

**Table 2:** Examples of inhibitors for chromatin-related proteins

Mode of action	Target	Compound name	Types of cancer	Reference
Enzymatic inhibition	DOT1L	EPZ-5676	MLL-rearranged leukemia	[85, 86]
	EZH2	EPZ6438, GSK126, CPI-1205	Lymphoma, malignant rhabdoid tumor	[9, 77, 87]
	p300	C646, A-485	hematological malignancies and androgen receptor-positive prostate cancer	[88]
	HDACs	Vorinostat, romidepsin	CTCL	[89, 90]
	CARM1	EZM2302	Multiple myeloma	[91]
PPI disruption	Menin-MLL	MI-503, MI-463, M-525	MLL-rearranged leukemia	[82, 83]
	WDR5-MLL	OICR-9429	C/EBPα N-terminal leukemia	[84]
	LEDGF-MLL	CP65	MLL-rearranged leukemia	[92]
Competitive binding	BET family of BRD proteins	JQ1, I-BET, I-BET151	NUT midline carcinoma, MLL-rearranged leukemia	[93–95]
Protein degradation	BRD4	dBET1, dBET6, ARV-825, ARV-771, BETd-246	AML, T-ALL, Burkitt’s lymphoma, castration-resistant prostate cancer, TNBC	[96, 97]

Histone H3K36me3 is detected in the body of actively transcribed genes due to the association of the enzyme SET2 with the phosphorylated form of CTD of RNA Pol II [39]. The function of H3K36me2, implemented by ASH1L and the NSD1-3 family, is less well-understood. Recently, a potential crosstalk between H3K4me3 and H3K36me2 was shown to occur at the hub of LEDGF [98]. LEDGF directly interacts with Menin and MLL1 through its integrase-binding domain and is required for MLL1-dependent transcription and leukemic transformation [99, 100]. Meanwhile, LEDGF binds to dimethylated H3K36 through its PWWP domain [98, 101]. LEDGF has drawn increasing attention since studies have shown that LEDGF is essential in MLL-rearranged leukemia, but not hematopoiesis, which raised the therapeutic potential of targeting LEDGF effectively without general side effects in the hematopoietic system [102, 103]. Due to the multifaceted roles of LEDGF and its interactions with a plethora of proteins with divergent functions [99, 104, 105], whether its role during leukemogenesis is dependent on MLL1 needs to be determined. Limited success for treating MLL-rearranged leukemia has been gained through targeting LEDGF using CP65, a cyclic peptide used for the inhibition of HIV viral replication, since the same domain on LEDGF bind to both the HIV integrase and MLL1 [92]. Degrading LEDGF may be a new direction using proteolysis targeting chimera (PROTAC) technology [106] which will be discussed in later sections. H3K36 me3 can also prevent methylation by PRC2 of the nearby H3K27 residue on the same histone tail [107].

Histone H3K79 methylation mark implemented by DOT1L, the only enzyme responsible for the deposition, is on the globular domain of the histones correlated with active gene expression [2, 40, 108]. DOT1L is also the only enzyme catalyzing lysine methylation that has a methyltransferase distinct from a SET domain, and a demethylase for H3K79 has not been identified to date. DOT1L is found in a complex named DotCom with MLL translocation partners AF9, or its paralog ENL, and AF10 [109]. DOT1L activity also promotes breast cancer cell proliferation and metastasis [110]. The aberrant upregulation of H3K79 methylation in leukemia [111] led to the development and use of DOT1L inhibitor EPZ-5676 for the treatment of MLL-rearranged leukemia [85] which is currently under clinical investigation [86].

Histone H3K9 and H3K27 methylations are required for the formation of distinct forms of heterochromatin [112]. Histone H3K9me3 and H3K27me3 have been proposed to be the only true “epigenetic marks” since they have defined mechanisms for being heritable after DNA replication [112]. The deposition machineries of H3K9me3 and H3K27me3 share a distinct “write-and-read” mechanism with both enzymatic activity and the ability to bind and recognize the modification within the same enzyme or enzyme complex, thus allowing for a positive feedback loop. For H3K9me3, SUV39H1 contains both the write-and-read module (chromodomain and SET domain) [113] and the methyl-lysine recognition further promotes methylation activity [114]. HP1 proteins—HP1α (CBX5), HP1β (CBX1), and HP1γ (CBX3), contain the methyl-lysine-binding chromodomain [115] and perform important roles in heterochromatin formation. Methylation of histone H3 lysine 9 written by SUV39H1 creates a binding site for HP1 proteins, which in turn recruit more SUV39H1, and this mechanism contributes to the propagation of heterochromatin formation [116]. In the case of H3K27me3, EZH2 implements H3K27 methylation within the PRC2 complex, while the EED subunit recognized this methylation and allosterically further activates the SET domain of EZH2 [23, 117]. Similar to the distinct distributions of H3K4me1/2/3 by COMPASS family, H3K27me1/2/3 distributions throughout the genome are mutually exclusive to each other, with H3K27me3 mainly at promoters (especially at bivalent genes), H3K27me2 at intergenic regions, and H3K27me1 in the gene bodies of actively transcribed genes [9]. Because EZH2 and SUZ12 subunits of PRC2 are required for HP1α stability, the heterochromatin markers H3K27me3 and H3K9 methylation may cooperate to maintain heterochromatin protein 1α at chromatin highlighting the crucial crosstalks between H3K9me2/3 and H3K27me3 pathways of gene silencing [118]. EZH2 inhibitors are frequently used to prevent unwanted histone methylation of tumor suppressor genes when EZH2 is aberrantly expressed in cancer cells or mutated (gain of function, Y641 in the SET domain) [9, 77, 119]. Our recent study demonstrated that cancer cells harboring MLL3 PHD mutations are more sensitive to the depletion of EZH2, SUZ12, and EED in the PRC2 complex [10]. Harnessing the synthetic lethality and dependency of the MLL3-UTX-PRC2 regulatory axis is a promising therapeutic stratification for the use of EZH2 inhibitors (Fig. 2b).

In addition to the frequent mutations of a broad spectrum of histone modifiers, several mutations on histone tails (H3K27M, H3K36M and H3G34V/R) have been found to be associated with tumorigenesis in different types of cancers [120]. A common feature of the mutated “oncohistones” is that they all impede the deposition of the proper histone modification at the mutation site, or surrounding residues in the case of H3G34 mutation, leading to transcriptional reprogramming and tumorigenesis [120]. A recurrent single-nucleotide substitution resulting in H3.3K27M has been discovered in diffused intrinsic pontine gliomas (DIPGs) accompanied with a global loss of H3K27me3 and reduced PRC2 catalytic activity, but higher levels of H3K27 acetylation, making it promising for BRD4 inhibition therapy [9, 87] (Fig. 2c). Histone H3K36M mutation is found in chondroblastoma, head and neck squamous cell carcinoma, and colorectal cancer, while H3G34V/R mutations have been found in both glioma and bone cancers [120]. Despite the limited progress in understanding the roles of these mutated histones in cancer development, there is an unmet need to perform a comprehensive synthetic lethality study to investigate whether the tumors bearing certain mutations are more dependent on certain signaling pathways for the exploration of potential therapeutic strategies to more effectively tailor treatment regimens for patients.

Methylation of H4K20 is associated with both transcriptional activation and repression depending on methylation states. H4K20me1 catalyzed by PR-Set7 is associated with activation and marking points of origin for DNA replication [121, 122]. On the other hand, H4K20me2/3 catalyzed by SUV4-20H1/2 is associated with repression of transcription by maintaining pericentric and telomeric heterochromatin [121]. H4K20me2/3 methylation can enhance chromatin condensation in vitro [25]. Loss of H4K20me3 has been described as a hallmark of cancer [26]. Dynamic regulation of H4K20 methylation was recently reported in *C. elegans*, where a new subfamily of the Jumonji C (JmjC) histone demethylases, DPY-21, was found to convert H4K20me2 to H4K20me1 to control higher-order structure of the two female X chromosomes, promote chromosome compaction, and repress gene expression [27]. Whether the human counterpart, RSBN1, has a role in reduced H4K20me3 in human cancer remains to be investigated.

In addition to the versatile states of lysine methylation, arginine residues can also be modified via monomethylation and symmetric and asymmetric dimethylation (MMA, SDMA, and ADMA) by a subset of protein arginine methyltransferases (PRMTs) including PRMT1, CARM1, PRMT5, and PRMT6 [123, 124]. The removal of the arginine methylation can occur through its deimination to citrulline by PADI4 [21] (please refer to section “Other types of histone modifications” for further discussion). PRMTs methylate not only histone tails, but also a large number of non-histone substrates [123]. This should be taken into consideration when interpreting studies using PRMT inhibitors since the outcomes may be through affecting numerous signaling pathways regulated by the substrates of a particular PRMT member. Nevertheless, success has been made developing specific inhibitors for CARM1/PRMT4 for the treatment of multiple myeloma [125], which can methylate H3R17me2a and H3R26me2a involved in transcriptional activation [123].s

Despite the tremendous progress made discovering the families of histone methyltransferases, demethylases, and the mutations of histones in cancer, there is still much to be learned of the biological roles of these proteins and their interplay in different developmental stages and disease settings.

#### Acetylation

Acetylation is a reversible modification on the ε-amino group of lysine residues that is controlled by two groups of enzymes: histone acetyltransferases (HATs) [126] and histone deacetylases (HDACs) [91]. There are three major families of HATs in humans that are well-studied including GNAT (HAT1, GCN5, PCAF), MYST (Tip60, MOF, MOZ, MORF, HBO1), and p300/CBP [127]. Notably, HATs can also catalyze the acetylation of a broad range of non-histone proteins including tumor suppressors and oncogenes, namely p53, Rb, and Myc to regulate protein stability, DNA binding, protein-protein interaction, enzymatic activity, or protein localization [89]. Acetylation of the histone tails neutralizes the positively charged lysines, which has been suggested to disrupt the interaction between the tail and the negatively charged nucleosomal DNA to facilitate opening of chromatin to promote active transcription. Acetylated lysines on chromatin can also promote open chromatin by being bound by a variety of bromodomain-containing transcription factors, including those in chromatin remodeling complexes such as the BAF complex [128, 129].

The well-conserved mark H4K16ac reduces chromatin compaction in vitro [130] and is associated with more open chromatin in vivo [131]. Genetic studies in Drosophila have shown that when H4K16 has been changed to arginine, female flies are viable and only males die due to the special role of H4K16ac in promoting X chromosomal dosage compensation. Reduced H4K16ac is associated with a variety of cancers [26, 126] and may in some cases have prognostic value [28].

Acetylation on H3K27 is prominent at active promoters and, together with p300 and H3K4me1, marks active enhancers [29, 132]. Histone H3K27ac is deposited by CBP/p300 and serves in part to counteract Polycomb silencing since acetylation precludes methylation by PRC2 at this site [133]. Acetylation not only affects the charge and promotes structural changes of chromatin, but the acetyl group also functions as a signal recognized by bromodomain (BRD)-containing proteins (acetyl-lysine binding proteins) such as the bromodomain and extraterminal domain (BET) bromodomain proteins BRD2, BRD3, and BRD4 [128]. Mutation, aberrant expression, and gene fusions have been found in these proteins and implicate their roles in cancer development and progression [22, 128, 134].

Deacetylation of histones by the HDACs diminishes the accessibility of transcription factors by forming a closed chromatin conformation [135]. There are 18 HDACs in mammals divided into four major families: Class I (HDACs 1, 2, 3, and 8) are ubiquitously expressed in human cell lines and tissues in the nucleus; Class II (HDACs 4, 5, 6, 7, 9 and 10) exhibit tissue-specific expression and can shuttle between the nucleus and cytoplasm; Class III or sirtuins (SIRT1-7), which are NAD^+^ dependent and have a very distinct catalytic mechanism for deacetylation compared with other classes of HDACs; Class IV has only one recently identified member, HDAC11 [136]. HDAC11 is capable of deacetylating divergent histone sites, making the substrate specificity low and functionally redundant in certain scenarios [3]. Similar to HATs, HDACs also have a number of non-histone substrates such as p53, Hsp90, TCF, and β-catenin [89].

Due to the dynamic nature of histone acetylation, inhibitors targeting HDACs, HATs, and bromodomain proteins have been developed and are in different preclinical and clinical stages for cancer therapy. Overexpression of HDACs has been found in a variety of cancers and correlates with significant decrease in both disease-free and overall survival and predicts poor patient prognosis [136–138]. HDAC activity is a key mediator of survival and tumorigenic capacity, making it a compelling target for a panel of different cancers, and indeed, HDAC inhibitors are the most mature epigenetic drugs developed to date. Vorinostat and romidepsin are FDA-approved HDAC inhibitors for the treatment of refractory cutaneous T cell lymphoma (CTCL), and there are many others currently under different stages of clinical assessment, most of them focused on hematological malignancies [138]. It should be noted that some of the HDAC inhibitors also exhibit inhibition activities towards PI3K (CUDC-907), EGFR (CUDC-101), and others. This may be desirable from the clinical perspective for limiting the dosage and toxicity by dually targeting two oncogenic pathways. However, it is a caveat when using this compound to study the molecular function of HDACs since they may exert efficacies through signaling pathways other than histone deacetylation. Despite the huge success targeting HDACs in the clinic, targeting HATs has lagged behind. C646 [139] and A-485 [90] are the only relatively potent and selective synthetic inhibitors for p300/CBP based on the virtual screening using a p300 HAT/Lys-CoA crystal structure. Their efficacy in preclinical models needs to be rigorously established in future studies.

The BET-bromodomain proteins are extensively studied, benefiting greatly from the availability of selective inhibitors [88, 140]. The strong phenotypic changes by BET protein inhibition justify the discovery and development of BET inhibitors to diminish their functions in hematological and solid tumors. The BET-bromodomain-specific inhibitors, JQ1 [141], I-BET [142], and I-BET151 [93], represent the initial successes of BET inhibitor development. The initial success of BET-bromodomain degraders has led to a series of studies increasing the potency of the compounds by linking the BET inhibitor moiety to the ligand that recruits the E3 ligase using the PROTAC technology [106] for degradation for the treatment of both hematologic disorders and solid tumors such as castration-resistant prostate cancer and triple negative breast cancer (TNBC) [94, 95, 143]. BET degraders, dBET1 and dBET6, potently and specifically target the BET bromodomain proteins for the treatment of AML and T-ALL [144, 145]. In this case, a phthalimide moiety is appended a competitive antagonist of BET bromodomains JQ-1 and the protein will undergo cereblon (CRBN) E3 ubiquitin ligase-dependent degradation [144]. Interestingly, studies have shown that thalidomide-targeted degradation can also be applied to selectively target the “undruggable” Zinc Finger (ZF) transcriptional factors using derivatized thalidomide analogs [96].

#### Ubiquitination

Monoubiquitination of histones most commonly occurs on H2A and H2B [97]. H2AK119 ubiquitination is implemented by RING1A/B in the PRC1 complex [146] and is removed by the BAP1 deubiquitinase complex [147]. H2AK119ub1 is linked with chromatin compaction and transcriptional silencing [146]. H2BK120ub1 is carried out by the UBE2A/B (RAD6) E2 ubiquitin conjugating enzyme and the RNF20/40 E3 ligase at actively transcribed genes [12]. The presence of H2BK120ub1 is coupled with high levels of methylation on H3K4 and H3K79 [13, 15, 148]. Similar to other modifications, histone ubiquitination is also linked with transcriptional activation and silencing by affecting a higher-order chromatin structure [97] and behaves as a signal for subsequent histone modifications via recruiting other machineries [149].

Crosstalk among epigenetic factors occurs at two major levels. First, numerous studies have demonstrated crosstalk between different histone modifications [3, 60]. For instance, histone H2B monoubiquitination is a prerequisite for H3K4 methylation by COMPASS, and H3K79 methylation by DOT1L [60]. On the contrary, H3K4 methylation by MLL/COMPASS inhibits the deposition of H3R2me2a by PRMT6, and vice versa, making the two marks mutually exclusive [3]. Second, the crosstalk between histone modifiers themselves can control normal and malignant states of cell proliferation. For instance, the BAP1 H2A deubiquitinase recruits H3K4 monomethylase MLL3 to monomethylate gene enhancers, while disruption of the interaction between BAP1 and MLL3 contributes to the pathogenesis of multiple cancers [10]. The H3K27 demethylase UTX is also a key component of MLL3/COMPASS, and its recruitment and activity is also dependent on BAP1 to execute proper functions at enhancers [10]. Other histone demethylases are also found in large histone-modifying complexes such as KMTs and HDACs. In this case, LSD1 is found in the CoREST-HDAC complex in association with HDACs, CoREST, and BHC80, and the interaction with these factors regulates its stability and activity [46].

#### Other types of histone modifications

Phosphorylation of histone tails adds a negative charge to the histone tails, thus changing the conformation of chromatin structure and interactions with transcription factors. Histone H3S10 phosphorylation is a well-characterized modification associated with chromosome condensation during mitosis and is implemented by the Aurora kinases, while H3S10p implemented by the MSK/Jil1 family is involved in positive regulation of transcription [150]. Dephosphorylation of this site is mediated by PP2A and is related to repression of gene expression [150]. Phosphorylation on Serine 139 of H2AX (γH2AX) is induced by stimuli of DNA damage and is an early response in DNA double-strand break signaling. Multiple kinases can mediate the phosphorylation on this particular site including ATM, ATR, and DNA-PK [151]. Although these two modifications have been intensively used as markers of cell cycle progression and the DNA damage response, the consequences of the modification and downstream events remain largely unknown [20]. Serine 31 phosphorylation is unique to histone H3.3 and was originally identified to be localized adjacent to centromeres in metaphase chromosomes [14]. It is also a mitosis-specific marker different from H3 S10P and S28P in terms of timing and localization [14]. Banaszynski’s group recently found that the function of H3.3.S31P to promote p300 activity and enhancer acetylation in mESCs [152].

With extensive studies being focused on methylation, acetylation, ubiquitination, and phosphorylation of histones, a plethora of other modifications have also been reported for histones including lysine crotonylation, butyrylation, propionylation, tyrosine hydroxylation, biotinylation, neddylation, sumoylation, O-GlcNAc, ADP ribosylation, N-formylation, proline isomerization, and citrullination [31, 153–156].

With the use of an integrated, mass spectrometry-based proteomics approach, lysine crotonylation has been designated as a specific mark of active sex chromosome-linked genes in post-meiotic male germ cells via associating with active chromatin, including promoters and active enhancers [82]. Intriguingly, the YEATS domain proteins display high binding affinity for crotonyl-lysine, linking this modification to active transcription [157]. Two recent studies highlight the possibility of targeting YEATS domains in MLL-rearranged leukemia, potentially synergizing with BET and DOT1L inhibition [158, 159]. Besides crotonylation, butyrylation and propionylation are two other non-acetyl-lysine acylation modifications actively occupying gene promoters and exerting their functions in a similar fashion as histone acetylation [160–162].

Neddylation, the covalent conjugation of NEDD8, a ubiquitin-like protein, is deposited on histone H2A by the E3 ligase RNF168. The neddylation of H2A on K119 prevents ubiquitination at this site and results in decreased response to DNA damage, suggesting a role of the neddylation pathway to DNA damage repair [163]. In addition to histone ubiquitination and neddylation, histone H4 can also be modified with SUMO (small ubiquitin-related modifier) family proteins to mediate transcriptional repression through the recruitment of histone deacetylases and heterochromatin protein 1 (HP1) [164].

Biotinylation of lysines on histones has also been described as a rare modification [165], but it has not been widely studied and its biological significance is not well-established. Serine/threonine O-GlcNAcylation of epigenetic factors such as HCF1 and TET2 has been well-established [166, 167]. However, whether histones are modified by O-GlcNAc in vivo in mammalian cells remain debated [168, 169]. The occurrence of histone ADP ribosylation is universal on all core histones and histone H1. Despite its universal presence, the biological consequence is quite divergent on different lysines modified ranging from DNA repair, replication and transcription [170]. N-formylation of lysines of histones represents a noncanonical secondary modification that arises from oxidative DNA damage [171]. Since the modification also occurs on lysine residues, it may interfere with methylation or acetylation of the same residue and contribute to the pathophysiology of oxidative and nitrosative stress. Likewise, noncovalent proline isomerization of histone H3 influences the H3K36 lysine methylation to enhance transcription [172]. Finally, deimination or citrullination of arginine residues by PADI4 antagonizes arginine methylation by converting arginine or methylarginine to the nonconventional amino acid citrulline [173, 174]. Hypercitrullination can promote chromatin decondensation [175]. Intriguingly, citrullinated histone H3 may also function as a novel prognostic marker associated with exacerbated inflammatory response in patients with advanced cancer [176].

Overall, some of these more recently identified histone modifications could affect the conventional modifications such as methylation, acetylation, ubiquitination, and phosphorylation via competing with the same sites on histones for modification or through crosstalk through conferring a conformational change, thus altering the downstream signaling and gene expression regulation. The rarity of these modifications in the genome may indicate functions in fine tuning the conventional modifications in response to various circumstances such as DNA damage and oxidative stress. The crosstalk and biological consequences of these rare modifications need to be further characterized in future studies.

### Techniques for mapping and characterizing the modifications and their genome distribution

Identification of histone modifications has been greatly aided by the development of mass spectrometric techniques [154, 177–180]. Bottom-up, middle-down, and top-down strategies have their own advantages and challenges [181, 182]. Bottom-up mass spectrometry typically analyzes small peptides generated from trypsinization, which can provide the highest accuracy for identifying modifications. Top-down mass spectrometry attempts to identify the entire complement of modifications starting from an intact protein. Middle-down mass spectrometric analyzes larger peptides generated from rarer histone-cutting enzymes such as Glu-C. The Middle-down approach allows relatively high sensitivity compared to top-down, while still allowing identification of the complement of modifications on an entire histone tail, the location of the majority of histone modifications. By identifying which modifications occur on the same histone, potential synergistic or antagonistic effects of different modifications can be revealed [182].

The successes in identifying numerous histone modifications leave the challenge of identifying the function of these modifications. In genetically tractable organisms such as yeast and fruit flies, organisms have been generated where all of the histone gene copies have been replaced with a mutation of a modification site to an unmodifiable residue [183–185]. shRNA-mediated knockdown or CRISPR/Cas9 knockout of histone modifiers can be used to assess the function of a histone modifier. Knocking in mutations of the catalytic site of the enzyme can be used to determine whether the effects observed upon loss of the modifier is due to the loss of the histone modification or due to disruption of the macromolecular, multifunctional complexes in which some of these enzymes are found.

The functional consequences of histone modifications in different conditions or perturbations can be evaluated with such techniques as RNA-seq for quantification of mature transcripts, precision nuclear run-on sequencing (PRO-seq) [186] or native elongating transcript sequencing (NET-seq) [187] for quantification of nascent transcripts. Methylated DNA immunoprecipitation sequencing (MeDIP-seq) [188], MethylC-seq [189], and reduced representation bisulfite sequencing (RRBS-seq) [190] can be used to measure changes in DNA methylation. Assay for Transposase-Accessible Chromatin sequencing (ATAC-seq) [191], DNAse-seq [192], and Formaldehyde-Assisted Isolation of Regulatory Element sequencing (FAIRE-seq) [193] can be used to assess changes in accessibility of chromatin. The advances of single-molecule detection of posttranslational modifications on nucleosomes allow the detection of combinatorial modification states and genomic positions of nucleosomes [194].

The development of enzymatic inhibitors can be challenging for a variety of histone modifiers: first, proteins within an enzyme family can preserve sequence and structural similarities, which can hinder the ability to obtain specific small molecule inhibitors; second, a large number of chromatin-related proteins lack druggable pockets. The aforementioned ligand-dependent degradation of proteins, PROTAC [106], HaloPROTAC [195, 196], small molecule-assisted shutoff (SMASh) degraders [197], and dTAG [198], has all been used to route target proteins for proteasome-dependent degradation (Table 3), thus bypassing the need for an enzymatic therapeutic target [199]. The use of these technologies to degrade chromatin-related proteins will significantly advance our understanding of the roles of histone modifications and chromatin in normal biological processes, as well as aid the rational design of efficient and potent small molecules with therapeutic value.

**Table 3:** Comparison of strategies that selectively target proteins for degradation

Method	Rate of action (*t*_1/2_)	Customized or universal ligand	Reversibility	Genetic manipulation required	Real-time visualization	Toxicity in mouse model	Degradation mediator
Thalidomide-targeted degradation	< 1 h	Customized	Yes	No	No	No	CRL4a^CRBN^ RING E3 ubiquitin ligase complex
PROTACs	< 2 h	Customized	Yes	No	No	No	CRL4a^CRBN^ and CRL2^VHL^ E3 ligase
HaloPROTACs	4–8 h/1 h	Universal HaloPROTAC3/bestatin 1b	Yes	Yes, HaloTag7 / HaloTag fusion	Yes	N/A	CRL2^VHL^ E3 ligase and IAP E3 ligase
SMASh	N/A	Universal asunaprevir	Yes	Yes, self-cleaving NS3pro-NS4A fusion	N/A	No	NS3 protease from hepatitis C virus
dTAG	< 1 h	Universal dTAG ligand	Yes	Yes, FKBP12 (F36 V) fusion	No	No	CRBN-dependent E3 ligase
AID	< 1 h	Universal auxin (IAA)	Yes	Yes, AID tag fusion and Tir1 F-box protein expression	Yes	Yes	CRL1^Tir1^

### From 2D to 4D: capturing nucleosomes dynamics

Due to the dynamic nature of nucleosomes and chromatin structure, various approaches are required to explore this space. The “2D” represents the broad spectrum of histone modifications as discussed in this review, either acting alone or in combination with other modifications for synergistic, additive, or antagonistic effects to sophisticatedly regulate gene expression in a timely manner. The “3D” lies in how histone modifications affect the chromatin organization, higher-order structures of chromatin, and interactions of distal regulatory elements. The 3D structure can be captured by Hi-C, a comprehensive way to measure chromatin interactions across the human genome [200]. Although histone modifications and chromatin architecture are profiled in separate assays, researchers are actively making predictions and modeling of the chromatin organization such as chromatin interaction hubs and topologically associated domain (TAD) boundaries using cell type-specific histone marks [201, 202]. The integration of ChIP-seq and Hi-C datasets reveal important information of how chromatin organization could have an impact on gene regulation and how chromatin architecture can be predicted using ChIP-seq data [203]. Nonetheless, an experimental method combining histone marks ChIP-seq and Hi-C would be useful to directly address these questions. Super-resolution imaging using a three-dimensional stochastic optical reconstruction microscope (3D-STORM) is another approach to capture the 3D organization of chromatin in different epigenetic states and reveal structural details of chromatin [204]. The “4D” relies on the real-time monitoring of modification dynamics. This could be achieved by using acute degradation strategies such as HaloPROTAC or auxin-inducible degron (AID) tagging of histone modifiers [205, 206], real-time visualization of chromatin modifications with confocal and structured illumination microscopy [207], and fluorescent ligand labeling for direct visualization of chromatin factors using Halo-tag [208, 209] or SNAP-tag [210]. The acute degradation strategies have apparent advantages over commonly shRNA-mediated knockdown or CRISPR/Cas9-mediated knockout of histone modifiers, which takes several days to months, and phenomena may be due to secondary effects. The acute degradation strategies are much more specific with less off-target effects and capture early effects on chromatin when coupled with conventional ChIP-seq or ATAC-seq. For example, 60 min of auxin treatment in cells with AID tagging of PAF1 resulted in a major depletion of endogenous PAF1 protein, confirming the release of Pol II from promoter-proximal pausing was a direct consequence PAF1 loss [206].

### Future directions

Great effort has been devoted to understand the role of histone modifications and the enzymatic machinery involved in the implementation of these modifications during development and in disease, especially for cancer. Precise techniques are being developed for mapping the localization and function of histone modifications in the genome from population of cells to hopefully few or even single cells. The proteins that specifically bind histone modifications translate information to regulate gene expression by recruiting or removing other transcription factors. It is crucial to characterize the various functions of PTMs and their modifiers in human cancer. Nevertheless, capturing modification dynamics remains a challenging problem to study the function of histone modifications in vivo.

Development of assays and specific small molecule inhibitors (enzymatic/non-enzymatic) for targeting disease-related PTMs requires extensive knowledge based on the X-ray/Cryo-EM crystal structures of modifiers and modification-binding factors. The tool molecules or chemical probes will further elucidate the in vivo biological function of the key players on chromatin. The “quality” (specificity and potency) of the chemical probes and the thoughtful design of the experimental assays largely determine the outcome and interpretation of results. In addition, the identification of non-histone substrates is critical for defining the roles of the histone modifiers in order to develop more specific inhibitors targeting the desired pathway [76, 89].

It is interesting that histone modifiers often reside within large multi-protein complexes for proper function, such as MLL/COMPASS, PRC2, and HDAC complexes. Understanding how the key enzymes function with other subunits (e.g., activity and stability regulation) within the same complex will inform the design of small molecule disruptors of the protein complexes. The MLL-menin and MLL-WDR5 inhibitors fall within this class, and other interfaces between the catalytic domain and scaffolding proteins may be desirable targets to be harnessed for small molecule development with the gain of structure knowledge. Together with other approaches for targeting histone modifications such as enzymatic activity inhibition and small molecule degraders by PROTACs, chromatin-related proteins and modifications are considered as favorable drug targets, and a number of agents have been designed and used in different stages of clinical trials combined with currently available chemotherapies [6].

Reducing the toxic side effects of epigenetic drugs is a challenging issue when testing the agents in clinical trials [211]. The synthetic lethal approach is currently being explored to reduce toxic off-target side effects and to combat therapy resistance by targeting multiple genes using a combination of drug treatments. Moreover, histone modifications may potentially act as biomarkers in cancer diagnosis and prognostic predictors [26, 212]. The ultimate goal is to translate epigenetic therapy into the clinic for the treatment of cancers and tailor efficient strategies based on cancer types and epigenome alterations.
